# Type II Robert's Uterus Presenting as Long‐Standing Secondary Subfertility: Diagnosis Confirmed by Combined Laparoscopy and Hysteroscopy in a Bangladeshi Patient

**DOI:** 10.1002/ccr3.72777

**Published:** 2026-05-24

**Authors:** Iftekhar Ahmed Sakib, Ayesa Perveen, Tahmina Begum, Nishad Tasnim

**Affiliations:** ^1^ Department of Obstetrics and Gynecology Shaheed Suhrawardy Medical College and Hospital Dhaka Bangladesh; ^2^ Manikganj Medical College Manikganj Bangladesh

**Keywords:** Bangladesh, hysteroscopy, laparoscopy, Müllerian anomaly, Robert's uterus, secondary subfertility

## Abstract

Robert's uterus is a rare congenital Müllerian duct anomaly characterized by an asymmetric septum dividing the uterine cavity into a communicating and a blind hemicavity despite a normal external contour. Although fewer than 200 cases have been reported globally, the incremental novelty of individual case reports lies in unique clinical presentation and diagnostic challenges rather than rarity alone. We report a case of Type II Robert's uterus in a 30‐year‐old Bangladeshi woman presenting with long‐standing (12 years) secondary subfertility in the absence of classical symptoms such as dysmenorrhea or hematometra, which is uncommon. Initial hysterosalpingography suggested a unicornuate uterus with bilateral tubal obstruction. Advanced imaging modalities (MRI and 3D ultrasonography) were not performed due to limited availability and financial constraints, necessitating definitive evaluation by combined laparoscopy and hysteroscopy. This revealed a normal external uterine contour, an asymmetric septate uterus with a blind left hemicavity, right tubal patency, and unilateral tubal obstruction. This case highlights two key contributions: (1) the potential for prolonged asymptomatic secondary subfertility in Type II Robert's uterus, and (2) the diagnostic value of combined endoscopy in the absence of advanced imaging in low‐resource settings. Early recognition is essential to avoid misdiagnosis and enable appropriate fertility‐preserving management.

## Key Clinical Message

Robert's uterus, a rare Müllerian anomaly, can closely mimic a unicornuate uterus on routine imaging and lead to misdiagnosis in infertile women. Combined diagnostic laparoscopy and hysteroscopy are essential for accurate identification, enabling appropriate fertility counseling and timely corrective management, particularly in low‐resource settings.

## 1 Introduction

Congenital Müllerian duct anomalies (MDAs) are caused by defects in the development, fusion, or resorption of the paired Müllerian ducts during embryogenesis. These anomalies affect 5%–7% of women in the general population and are more prevalent in women with infertility, recurrent miscarriages, and adverse obstetric outcomes [1, 2]. Accurate diagnosis is crucial because treatment and reproductive prognosis vary significantly depending on the anomaly type.

Robert's uterus is an extremely rare form of septate uterus, first described in 1970 [3]. It is characterized by a complete asymmetric septum dividing the uterine cavity into a communicating hemicavity and a blind, non‐communicating hemicavity, while maintaining a normal external uterine contour [1, 4]. This often leads to misdiagnosis as a unicornuate uterus or uterus didelphys [4, 5].

Despite its rarity, this case is distinguished by an unusually prolonged duration of secondary subfertility (12 years), absence of classical symptoms, and reliance on combined endoscopic diagnosis in a resource‐limited setting. Here, we present a case of Type II Robert's uterus diagnosed using combined laparoscopy and hysteroscopy in a Bangladeshi patient.

## 2 Case Presentation

A 30‐year‐old woman presented to the gynecology outpatient department with a history of secondary subfertility for 12 years. She had been married for 15 years and had conceived twice previously. Her menstrual history was unremarkable, with regular cycles occurring every 28 days and lasting four to five days. She denied dysmenorrhea, menorrhagia, intermenstrual bleeding, or cyclical pelvic pain. Her obstetric history included one full‐term normal vaginal delivery and one cesarean section, with a history of one perinatal death. Her last living child was 13 years old. There were no reported antenatal, intrapartum, or postpartum complications during her previous pregnancies. She had no significant medical or surgical history and was not on any long‐term medications. On general examination, the patient was hemodynamically stable, and systemic examination revealed no abnormalities. Abdominal and pelvic examinations were unremarkable, with no palpable masses or tenderness. Routine laboratory investigations were within normal limits, and her blood group was O negative.

## 3 Methods

### 3.1 Differential Diagnosis

The differential diagnoses considered included:

1. Unicornuate uterus with non‐communicating horn.
2. Uterus didelphys.
3. Septate uterus.

### 3.2 Investigations

**FIGURE 1:**
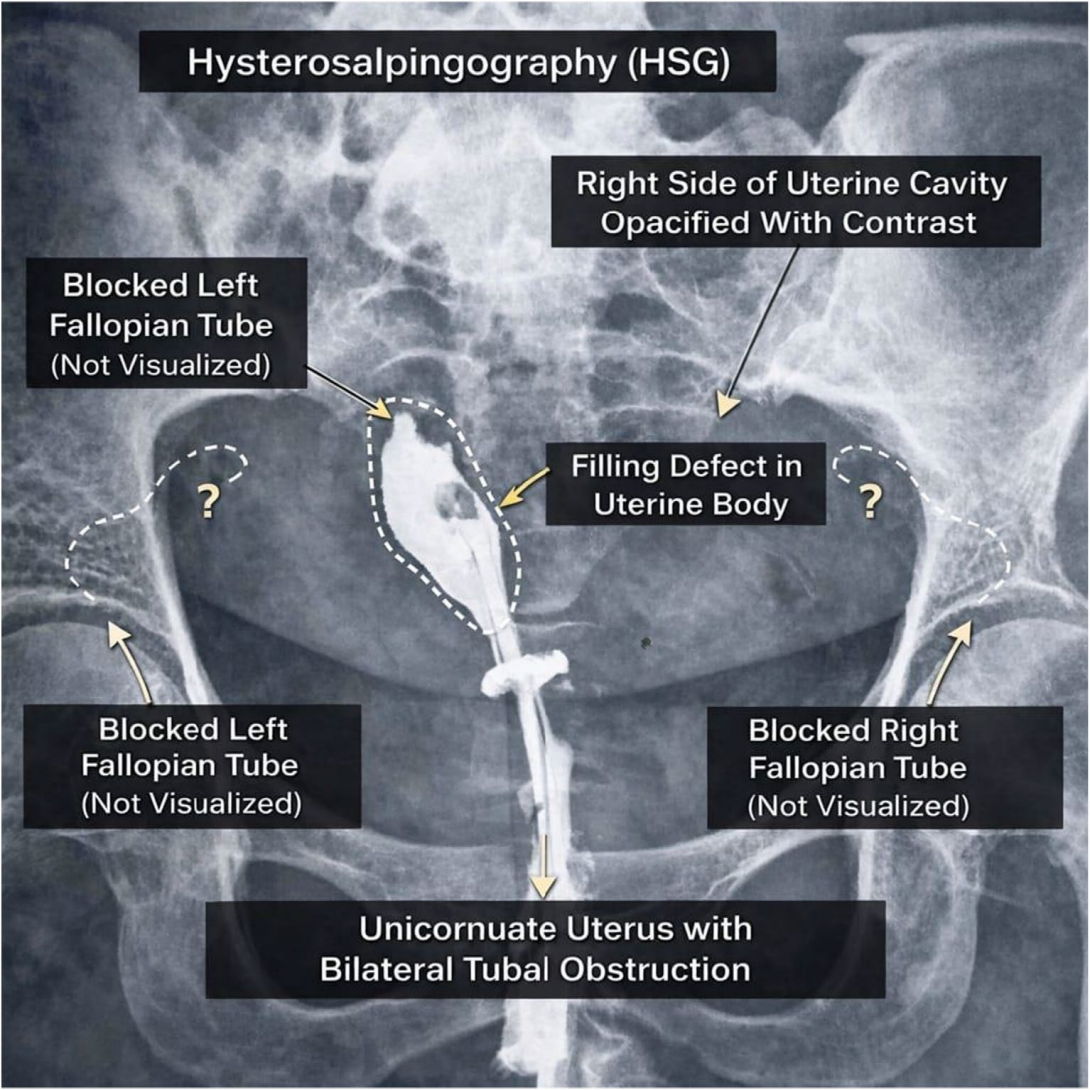
Hysterosalpingography Demonstrating Unicornuate Uterus with Bilateral Tubal Obstruction. Hysterosalpingogram (HSG) showing opacification of only the right side of the uterine cavity following transcervical injection of contrast medium, consistent with a unicornuate uterus. A filling defect is noted within the uterine body. Both fallopian tubes are not visualized, indicating bilateral tubal obstruction. No free peritoneal spillage of contrast is observed. The control film shows no radio‐opaque shadow in the pelvic region. These findings are suggestive of a unicornuate uterus with bilateral tubal obstruction.

As part of the subfertility workup, hysterosalpingography was done. Hysterosalpingography findings are shown in Figure 1, which suggest a unicornuate uterus with bilateral tubal obstruction.

Three‐dimensional ultrasonography and MRI were not performed due to limited availability and financial constraints in the patient's setting, and because endoscopic evaluation was considered a definitive diagnostic approach.

Given the prolonged duration of subfertility and the inconclusive nature of imaging, combined diagnostic laparoscopy and hysteroscopy were planned for definitive evaluation.

**FIGURE 2:**
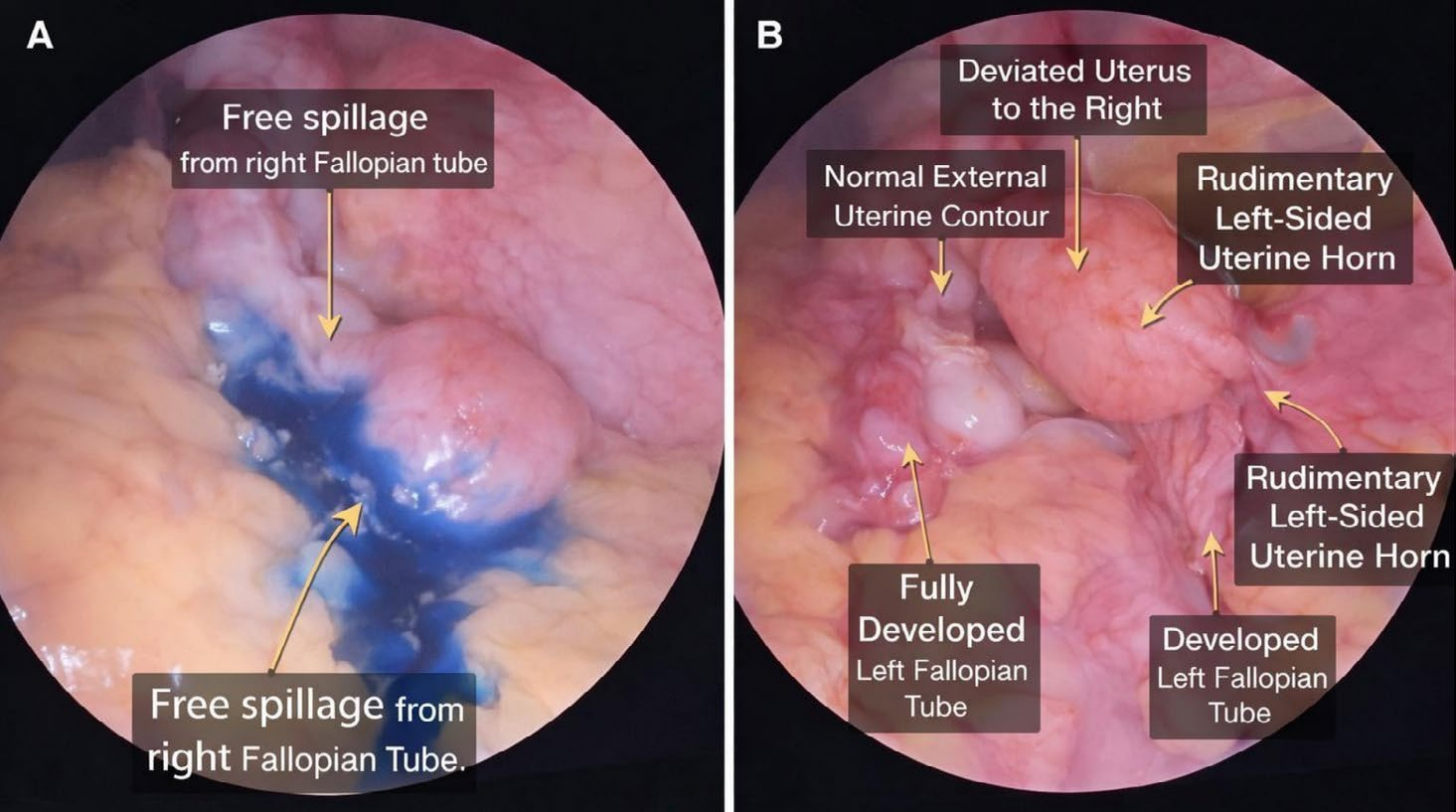
Diagnostic Laparoscopy Findings in Type II Robert's Uterus. (A) Chromopertubation demonstrating free spillage of blue dye from the right fallopian tube, confirming right tubal patency. (B) Laparoscopic view showing a uterus deviated to the right with a normal external contour and a rudimentary left‐sided uterine horn. No dye spillage is observed from the left fallopian tube.

Under general anesthesia, the patient had a combined diagnostic laparoscopy and hysteroscopy. Pelvic adhesions were discovered during laparoscopic examination and were removed through adhesiolysis. The uterus appeared deviated to the right but had a normal external contour. A rudimentary left‐sided uterine structure was observed, and the left fallopian tube was well developed. A left ovarian cyst was also identified and excised by complete cystectomy. Intraoperative laparoscopic findings (Figure 2A,B) demonstrated right tubal patency and the absence of left‐sided dye spillage.

**FIGURE 3:**
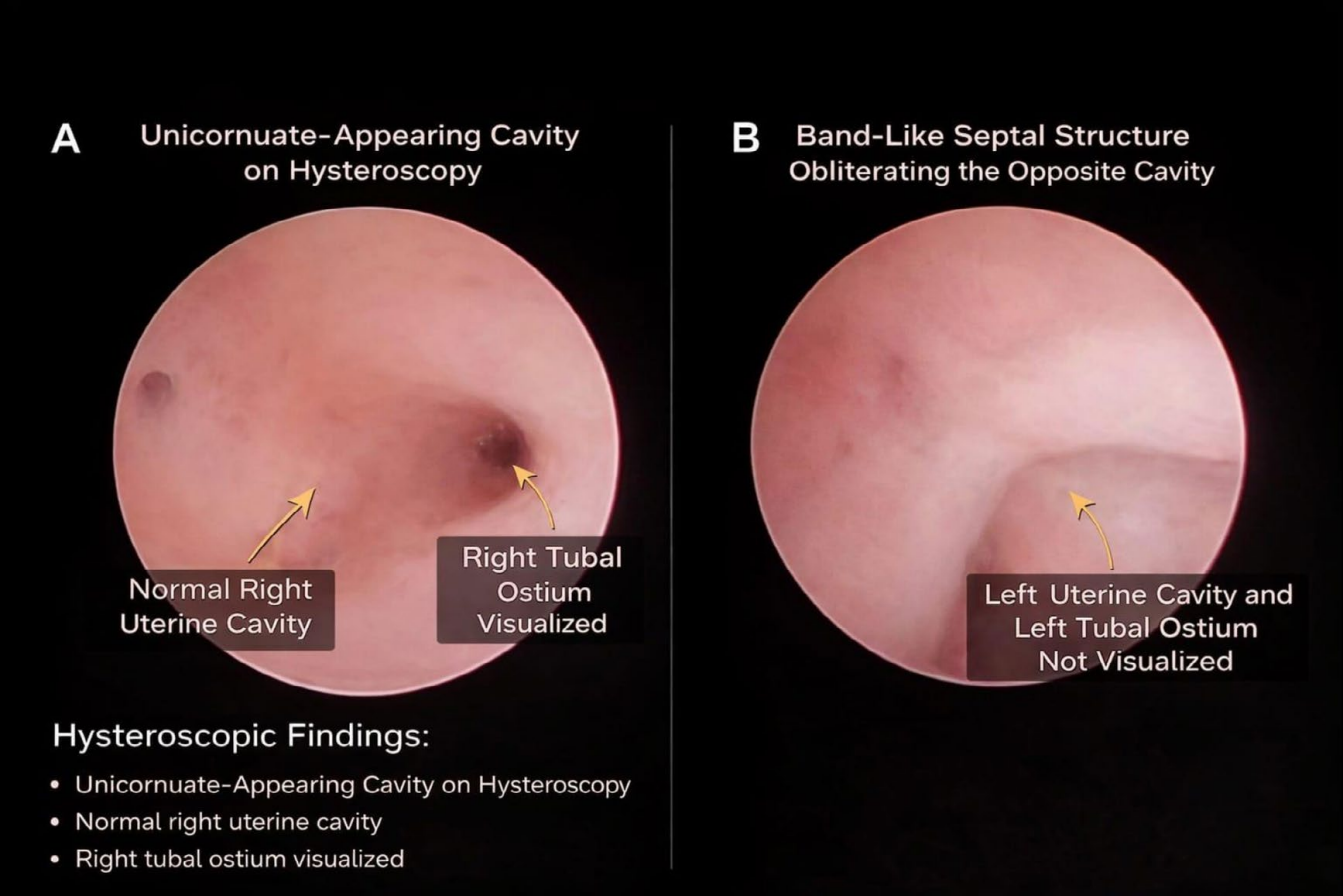
Hysteroscopic Views in a Case of Robert's Uterus. (A) Hysteroscopic view showing an asymmetrical uterine cavity with a blind, non‐communicating hemi‐cavity separated by a longitudinal septum. (B) Hysteroscopic view of the communicating hemi‐cavity with normal endometrium and a visible septal partition.

Hysteroscopic findings (Figure 3A,B) revealed a single communicating cavity with a septal obstruction. The right uterine cavity was normal, and the right tubal ostium was visualized. The left uterine cavity and left tubal ostium were not visualized due to a thick, band‐like septal structure obliterating the contralateral cavity.

Type II Robert's uterus with a right‐sided functional cavity, blind left hemi‐cavity, unilateral tubal obstruction, and a left ovarian hemorrhagic corpus luteum cyst was the ultimate intraoperative diagnosis. A hemorrhagic corpus luteum without cancer was confirmed by histopathology.

### 3.3 Treatment Summary

1. Adhesiolysis
2. Ovarian cystectomy
3. Planned (but deferred): hysteroscopic septal resection (metroplasty)

## 4 Results (Outcome and Follow‐Up)

The patient was diagnosed intraoperatively with Type II Robert's uterus. Postoperatively, she remained stable and was counseled regarding fertility‐preserving surgical options, including hysteroscopic septal resection (metroplasty). Due to financial limitations and personal considerations, immediate corrective surgery was deferred. The patient was advised regarding assisted reproductive options and scheduled for follow‐up; however, long‐term reproductive outcomes are currently unavailable.

## 5 Discussion

### 5.1 Embryological Basis of Robert's Uterus

Robert's uterus results from incomplete and asymmetric resorption of the uterovaginal septum following normal Müllerian duct fusion. This embryological defect leads to the formation of a single uterine body with a normal external fundal contour but an internally divided uterine cavity consisting of a functional hemi‐cavity that communicates with the cervix and a contralateral blind, non‐communicating hemi‐cavity [1, 2, 3]. The preservation of a normal external uterine contour distinguishes Robert's uterus from fusion defects and is a major contributor to diagnostic confusion on conventional imaging modalities.

### 5.2 Classification of Robert's Uterus

**FIGURE 4:**
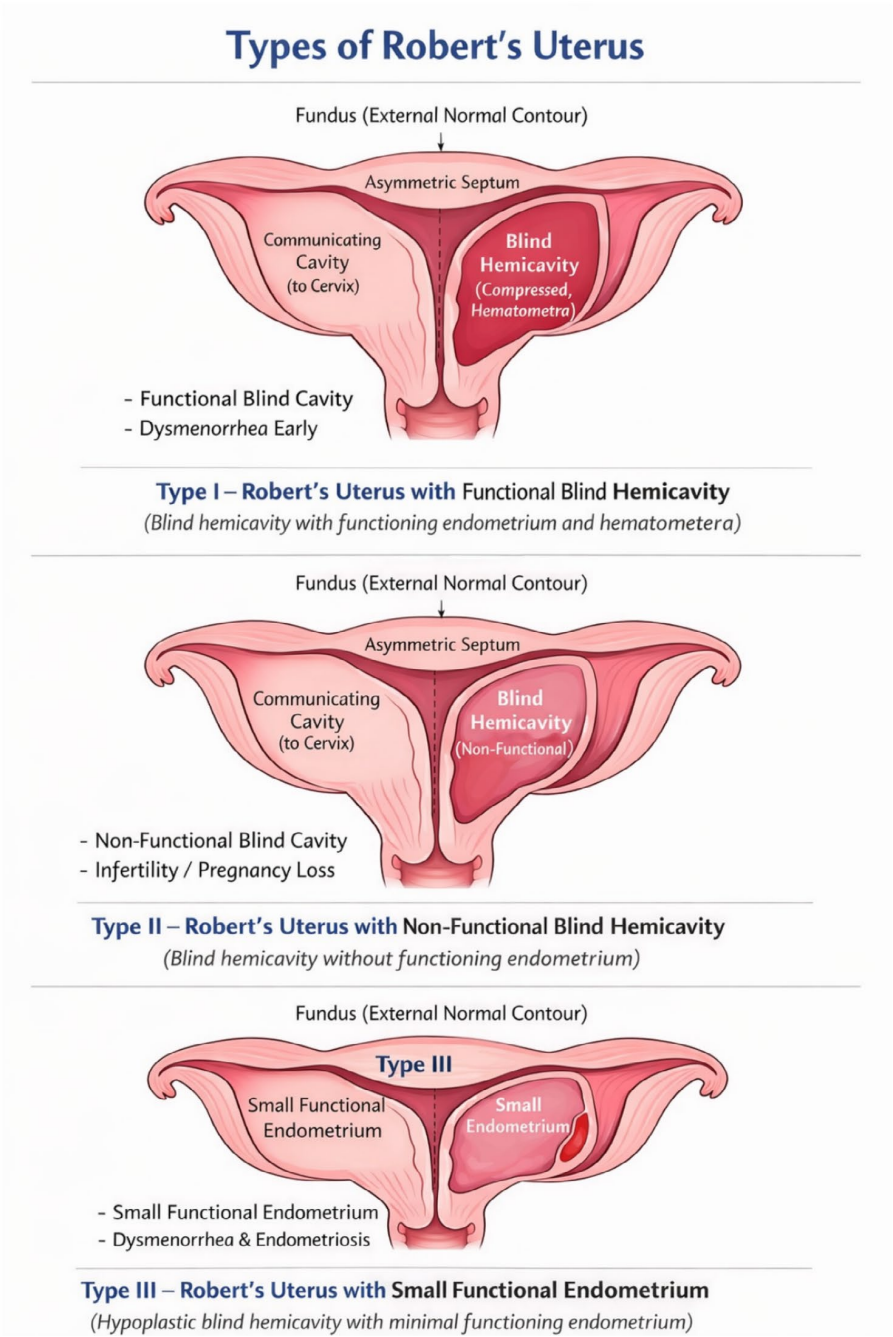
Schematic Diagram Demonstrating Morphological Types of Robert's Uterus. Type I (typical Robert's uterus) showing a functional blind hemicavity with hematometra causing compression of the contralateral communicating hemicavity. This type is commonly associated with early‐onset dysmenorrhea. Type II showing a non‐functional blind hemicavity with a normally functioning communicating hemicavity. This type is more frequently associated with infertility or recurrent pregnancy loss. Type III showing a blind hemicavity containing a small amount of functional endometrium, which may result in limited hematometra and is often associated with dysmenorrhea and secondary endometriosis. The schematic illustrates the three morphological types of Robert's uterus, a rare asymmetric septate uterus characterized by a normal external fundal contour and a complete asymmetric uterine septum dividing the uterine cavity into a communicating hemicavity and a non‐communicating (blind) hemicavity.

As shown in Figure 4, Robert's uterus has been classified into three morphological types based on the functional status of the blind hemicavity [3, 5].

### 5.3 Mechanisms of Subfertility in Type II Robert's Uterus

**FIGURE 5:**
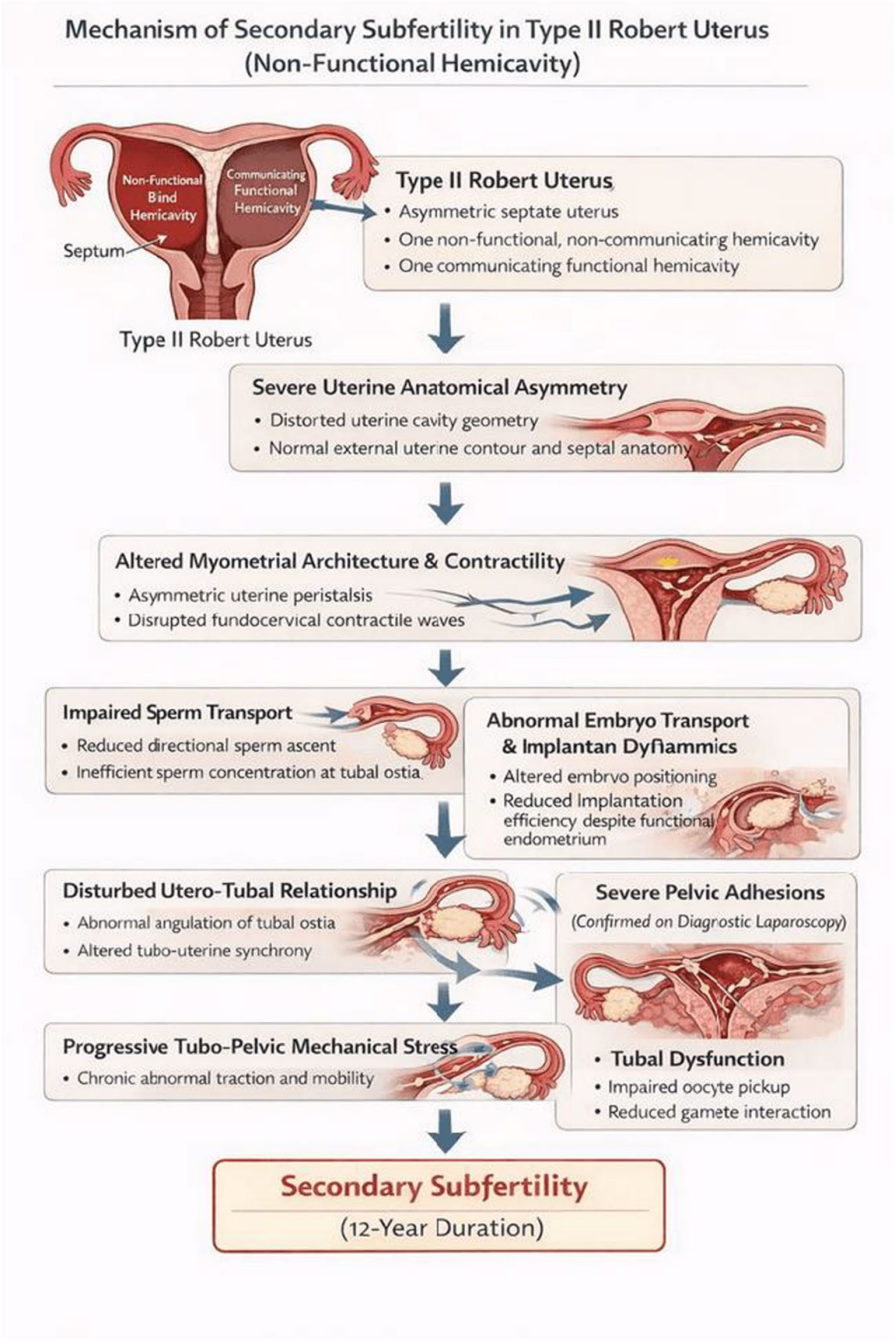
Schematic Diagram Illustrating the Mechanism of Secondary Subfertility in Type II Robert's Uterus. Schematic diagram demonstrating the proposed pathophysiological mechanisms leading to secondary subfertility in Type II Robert's uterus with a non‐functional blind hemi‐cavity and a normal external uterine contour. The diagram illustrates abnormal intrauterine anatomy, asymmetric uterine septation, altered uterine cavity dynamics, impaired sperm transport, disrupted embryo implantation, and associated endometrial and myometrial dysfunction contributing to reduced fertility.

Although Type II Robert's uterus lacks hematometra and overt obstructive symptoms, it may significantly impair fertility. The marked asymmetry of the uterine cavity and altered myometrial architecture can result in abnormal uterine peristalsis, impaired sperm transport, disrupted embryo migration, and reduced implantation efficiency within the functional hemicavity [6]. Additionally, distortion of the utero‐tubal relationship may predispose to progressive tubo‐pelvic adhesions, as observed in this patient, further contributing to long‐standing secondary subfertility despite the absence of endometriosis or cyclical pain as shown in Figure 5.

Figures 4 and 5 are schematic representations created by the authors for illustrative purposes. No copyrighted material has been reproduced.

### 5.4 Diagnostic Challenges

Hysterosalpingography and two‐dimensional ultrasonography frequently misclassify Robert's uterus as a unicornuate uterus because only a single hemicavity is visualized, often accompanied by apparent tubal non‐visualization or false tubal obstruction [4, 7]. The discrepancy between HSG and laparoscopic findings (bilateral obstruction vs. unilateral patency) is likely explained by false‐negative HSG results, possibly due to tubal spasm, technical factors, or distortion of the uterine cavity by the asymmetric septum. This represents an important diagnostic pitfall. This diagnostic pitfall is particularly consequential in low‐ and middle‐income countries (LMICs), where Robert's uterus is exceedingly rare, clinician familiarity is limited, and diagnostic pathways rely heavily on conventional imaging. Recent advances in imaging have significantly improved the diagnostic accuracy of Müllerian anomalies. Comparative studies demonstrate that magnetic resonance imaging (MRI) provides superior anatomical delineation with high diagnostic accuracy (AUC ~0.92), while three‐dimensional ultrasonography (3D‐US) also shows excellent performance and strong concordance with MRI in many cases. However, despite these advances, both modalities may still face limitations in complex or atypical anomalies, particularly when uterine anatomy is markedly asymmetric [8, 9]. Recent evidence supports the superiority of combined endoscopic evaluation in complex Müllerian anomalies, particularly when imaging findings are inconclusive [10].

### 5.5 Clinical Significance and Novel Insight

Unlike classical presentations associated with dysmenorrhea or hematometra, this case demonstrates that Type II Robert's uterus may remain clinically silent and present solely with long‐standing secondary subfertility. This underscores the need for high clinical suspicion even in asymptomatic patients.

### 5.6 Review of Literature and Fertility Outcomes

Robert's uterus is an exceptionally rare asymmetric Müllerian duct anomaly, with fewer than 200 cases reported worldwide, predominantly from Europe and East Asia [7, 11]. Due to its normal external uterine contour and unilateral cavity visualization on conventional imaging, it is frequently misdiagnosed as a unicornuate uterus, particularly when hysterosalpingography or two‐dimensional ultrasonography is used alone [4, 12]. Reported misdiagnosis rates approach 40%–50% in published series [4].

### 5.7 South Asian Perspective

In South Asia, patients may receive inappropriate fertility counseling, delayed interventions, or unnecessary surgical procedures due to the misdiagnosis explained above [13]. This case represents a rare documented report from Bangladesh, emphasizing the importance of clinician awareness and the role of combined hysteroscopic and laparoscopic evaluation to achieve accurate diagnosis when advanced imaging is not feasible [12, 14]. The use of these endoscopic modalities allows for direct visualization of both the intracavitary anatomy and external uterine contour, which is essential for distinguishing Type II Robert's uterus from other Müllerian anomalies.

### 5.8 Management Considerations

Management should be individualized based on the patient's symptoms and reproductive goals. For women desiring fertility, hysteroscopic septal resection or unification metroplasty is recommended to restore a single functional uterine cavity and improve reproductive outcomes [15, 16]. In cases where conception does not occur postoperatively, assisted reproductive technologies (ART) may be considered as adjunctive treatment [15, 17]. Early recognition and timely intervention are therefore critical to optimize reproductive outcomes, prevent long‐term complications such as secondary infertility or pelvic adhesions, and reduce the psychosocial burden associated with delayed diagnosis [18, 19].

## 6 Limitations

This report is limited by:

1. Single‐case design.

2. Absence of MRI/3D imaging confirmation.

3. Lack of long‐term reproductive follow‐up.

4. Mechanistic explanations based on literature rather than direct evidence.

## 7 Conclusions

Robert's uterus represents one of the rarest forms of Müllerian duct anomalies and poses a significant diagnostic challenge. The Type II variant, characterized by a non‐functional, non‐communicating hemicavity, may remain clinically silent for years and present later with long‐standing secondary subfertility, in the absence of classical symptoms such as dysmenorrhea or hematometra.

This case contributes novel insight by demonstrating:

– Prolonged asymptomatic secondary subfertility (12 years).
– Diagnostic discrepancy between HSG and laparoscopy.
– Feasibility of endoscopic diagnosis in absence of advanced imaging.

Accurate intraoperative identification not only prevents misdiagnosis as a unicornuate uterus but also has critical implications for fertility counseling and management. As one of the few documented cases from Bangladesh, this report contributes to the limited South Asian literature on Robert's uterus and underscores the need for increased clinician awareness of rare asymmetric Müllerian anomalies. Early recognition and appropriate endoscopic evaluation are essential to avoid inappropriate interventions, guide fertility‐preserving surgical options, and ultimately optimize reproductive outcomes for affected women.

## Author Contributions

**Iftekhar Ahmed Sakib:** conceptualization, data curation, formal analysis, investigation, methodology, project administration, resources, software, supervision, validation, visualization, writing – original draft, writing – review and editing. **Ayesa Perveen:** conceptualization, data curation, formal analysis, investigation, methodology, project administration, resources, supervision, validation, visualization, writing – review and editing. **Tahmina Begum:** conceptualization, data curation, formal analysis, investigation, methodology, project administration, resources, supervision, validation, writing – review and editing. **Nishad Tasnim:** data curation, formal analysis, investigation, methodology, project administration, resources, visualization, writing – original draft, writing – review and editing.

## Funding

The authors have nothing to report.

## Consent

Written informed consent was obtained from the patient for publication of this case report and accompanying images.

## Conflicts of Interest

The authors declare no conflicts of interest.

## Supporting information


**Video S1:** Combined diagnostic laparoscopy and hysteroscopy demonstrating intraoperative findings of Type II Robert's uterus with unilateral tubal patency and asymmetric septate cavity.
**Figure 6:** Placeholder Image for Video S1. Video S1 demonstrating combined laparoscopic and hysteroscopic findings in Type II Robert's uterus. The video shows a normal external uterine contour with right‐sided deviation, unilateral right tubal dye spillage on chromopertubation, absence of left‐sided spillage, and hysteroscopic visualization of the communicating hemi‐cavity separated by an asymmetric septal structure.

## Data Availability

The data that support the findings of this study are available from the corresponding author upon reasonable request. The data are not publicly available due to privacy and ethical restrictions related to patient confidentiality.
